# Influenza Activity and Composition of the 2022–23 Influenza Vaccine — United States, 2021–22 Season

**DOI:** 10.15585/mmwr.mm7129a1

**Published:** 2022-07-22

**Authors:** Angiezel Merced-Morales, Peter Daly, Anwar Isa Abd Elal, Noreen Ajayi, Ekow Annan, Alicia Budd, John Barnes, Arielle Colon, Charisse N. Cummings, A. Danielle Iuliano, Juliana DaSilva, Nick Dempster, Shikha Garg, Larisa Gubareva, Daneisha Hawkins, Amanda Howa, Stacy Huang, Marie Kirby, Krista Kniss, Rebecca Kondor, Jimma Liddell, Shunte Moon, Ha T. Nguyen, Alissa O’Halloran, Catherine Smith, Thomas Stark, Katie Tastad, Dawud Ujamaa, Dave E. Wentworth, Alicia M. Fry, Vivien G. Dugan, Lynnette Brammer

**Affiliations:** 1 Influenza Division, National Center for Immunization and Respiratory Diseases, CDC.

Before the emergence of SARS-CoV-2, the virus that causes COVID-19, influenza activity in the United States typically began to increase in the fall and peaked in February. During the 2021–22 season, influenza activity began to increase in November and remained elevated until mid-June, featuring two distinct waves, with A(H3N2) viruses predominating for the entire season. This report summarizes influenza activity during October 3, 2021–June 11, 2022, in the United States and describes the composition of the Northern Hemisphere 2022–23 influenza vaccine. Although influenza activity is decreasing and circulation during summer is typically low, remaining vigilant for influenza infections, performing testing for seasonal influenza viruses, and monitoring for novel influenza A virus infections are important. An outbreak of highly pathogenic avian influenza A(H5N1) is ongoing; health care providers and persons with exposure to sick or infected birds should remain vigilant for onset of symptoms consistent with influenza. Receiving a seasonal influenza vaccine each year remains the best way to protect against seasonal influenza and its potentially severe consequences.

The United States influenza surveillance system is a collaborative effort between CDC and its many partners in state, local, and territorial health departments, public health and clinical laboratories, vital statistics offices, health care providers, hospitals, clinics, emergency departments, and long-term care facilities. This report is a summary of the 2021–22 influenza season. This report was reviewed by CDC and was conducted consistent with applicable federal law and CDC policy.^†^

## Virus Surveillance

**FIGURE 1:**
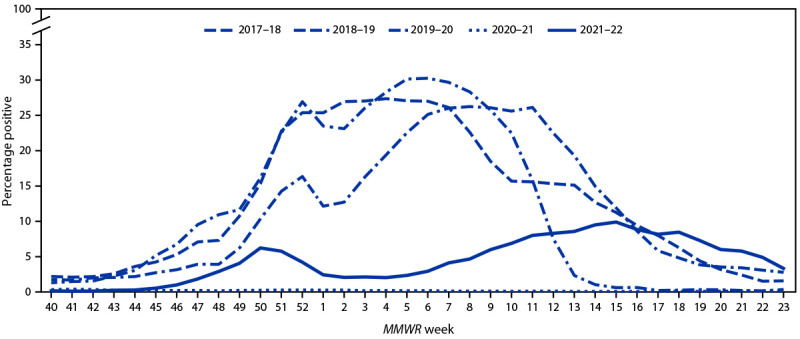
Influenza-positive test results reported by clinical laboratories to CDC, by *MMWR* week and influenza season — United States, October–June, 2017–18 to 2021–22

U.S. World Health Organization (WHO) collaborating laboratories and National Respiratory and Enteric Virus Surveillance System laboratories, which include both clinical and public health laboratories throughout the United States, contribute to virologic surveillance for influenza. Clinical laboratories tested 2,850,954 respiratory specimens using clinical diagnostic tests for influenza viruses. Among these, 128,302 (4.5%) specimens tested positive, including 126,477 (98.6%) for influenza A and 1,825 (1.4%) for influenza B. The percentage of specimens testing positive for influenza each week ranged from 0.1% to 9.9% (Figure 1). Public health laboratories tested 877,928 specimens and reported 24,432 (2.8%) positive specimens, with 24,306 (99.5%) positive for influenza A and 126 (0.5%) positive for influenza B viruses. Among 19,127 seasonal influenza A viruses that were subtyped, 25 (0.1%) were influenza A(H1N1)pdm09 viruses, and 19,102 (99.9%) were influenza A(H3N2) viruses. Influenza B lineage information was available for 41 (32.5%) influenza B viruses; 40 (97.6%) were B/Victoria lineage viruses, and one (2.4%) was a B/Yamagata lineage virus.^§^ Influenza A(H3N2) was the predominant virus throughout the 2021–22 influenza season nationally and among all 10 U.S. Health and Human Services (HHS) regions.**^¶^** The percentage of specimens testing positive for influenza in clinical laboratories had two distinct waves in nine of the 10 HHS regions; region 8 (Mountain) experienced a single wave of influenza activity. These nine regions experienced a first wave that peaked in mid-December 2021. A second wave occurred later with peaks ranging from mid-March to May 2022. Regions 6 and 7 (Central and South Central, respectively) peaked in mid-March; regions 2, 3, and 5 (New York/New Jersey/Puerto Rico, mid-Atlantic, and Midwest, respectively) peaked in April 2022; and regions 1, 4, 8, 9, and 10 (New England, Southeast, Mountain, West Coast, and Pacific Northwest, respectively) peaked in May 2022. All 10 regions experienced the highest percentage of positive test results during the later time frame.

Among the A(H3N2) viruses with age data available, 10%, 51%, 28%, and 11% were reported from persons aged 0–4, 5–24, 25–64, and ≥65 years, respectively. The number of A(H1N1)pdm09, B/Victoria, and B/Yamagata viruses reported was too small to analyze by age group.

## Novel Influenza A

Novel influenza viruses are influenza A virus subtypes that are different from currently circulating human seasonal influenza H1 and H3 viruses. During the 2021–22 influenza season, four novel influenza A viruses were detected in humans. Three were variant viruses (i.e., a swine influenza virus identified in a person and designated with a “v”); one A(H1N2)v virus was identified in a person in California, one A(H3N2)v in a person in Ohio, and one A(H1)v in a person in Oklahoma. One avian A(H5N1) virus was identified in a person in Colorado who was exposed to birds infected with highly pathogenic avian influenza A(H5N1). The A(H5N1) identification was the first positive test result for avian influenza A(H5) virus in a human in the United States (*1*).

## Virus Characterization

Genetic characterization was carried out using next-generation sequencing, and the genomic data were analyzed and submitted to public databases (GenBank: https://www.ncbi.nlm.nih.gov/genbank/ or EpiFlu: https://www.gisaid.org/). Antigenic characterizations were conducted using hemagglutination inhibition assays or virus neutralization–based focus reduction assays to evaluate whether genetic changes in circulating viruses affected antigenicity; substantial differences could affect vaccine effectiveness. CDC genetically characterized 1,757 specimens (1,733 influenza A and 24 influenza B) collected in the United States during October 3, 2021–June 11, 2022, and antigenically characterized a subset of genetically characterized specimens (*2*). All 12 genetically characterized A(H1N1)pdm09 viruses belonged to the 6B.1A clade, with nine belonging to the 5a.1 subclade, and three belonging to the 5a.2 subclade. Of the five A(H1N1)pdm09 viruses antigenically characterized, two were well recognized by ferret antisera raised against cell-grown and egg-grown vaccine reference viruses. All 1,721 influenza A(H3N2) viruses genetically characterized belonged to the 3C.2a1b clade with 1,717 (99.7%) belonging to the 2a.2 subclade. Of the 129 A(H3N2) viruses antigenically characterized, five (3.9%) were well recognized by ferret antisera raised against cell-grown vaccine reference viruses, and 22 (17%) well recognized by ferret antisera raised against egg-grown vaccine reference viruses. Among the 24 influenza B/Victoria viruses that were tested, all belonged to the V1A clade, with 15 (62.5%) belonging to the V1A.3a.2 subclade and nine (37.5%) belonging to the V1A.3 subclade. Eleven (73%) of the 15 B/Victoria viruses antigenically characterized were well recognized by ferret antisera raised against cell-grown and egg-grown vaccine reference viruses.

CDC also analyzes influenza viruses for susceptibility to antivirals. A total of 1,782 viruses were genetically characterized for susceptibility to neuraminidase inhibitors, and a subset of 314 (19%) were tested phenotypically (*2*). All genetically characterized viruses were predicted to be susceptible to the neuraminidase inhibitors, except three A(H1N1)pdm09 viruses that had an NA-H275Y amino acid substitution, a marker of oseltamivir resistance. Among the viruses tested phenotypically, only three A(H1N1)pdm09 viruses that had an NA-H275Y amino acid substitution did not display normal inhibition by neuraminidase inhibitors. A total of 1,757 viruses were genetically characterized for susceptibility to the polymerase acidic (PA) cap-dependent endonuclease inhibitor baloxavir, and a subset of 535 (33%) were tested phenotypically (*2*). One A(H3N2) virus had a PA-I38M amino acid substitution, which conferred reduced baloxavir susceptibility, and all remaining tested viruses were susceptible to baloxavir.

## Composition of the 2022–23 Influenza Vaccines

Vaccine strains for the 2022–23 influenza vaccines were selected by the Food and Drug Administration’s Vaccines and Related Biologic Products Advisory Committee based on WHO’s recommended Northern Hemisphere 2022–23 influenza vaccine composition. No changes were made to the A(H1N1)pdm09 or the B/Yamagata egg-based, cell-based, or recombinant vaccine recommended components. The recommended A(H3N2) component was changed to an A/Darwin/9/2021 (H3N2)–like virus for egg-based vaccines and an A/Darwin/6/2021 (H3N2)–like virus for cell-based or recombinant vaccines. The B/Victoria component recommendation was changed to a B/Austria/1359417/2021–like virus (*3*,*4*). The clade and subclade for the recommended vaccine strains were 6b.1A.5a.2 for A(H1N1)pdm09, 3C.2a1b.2a.2 for A(H3N2), V1A.3a.2 for B/Victoria, and Y3 for B/Yamagata.

## Outpatient Illness Surveillance

Nationally, the weekly percentage of outpatient visits for respiratory illness that included fever plus a cough or sore throat, also referred to as influenza-like illness (ILI),** recorded in the U.S. Outpatient Influenza-like Illness Surveillance Network was at or above the national baseline^††^ (2.5%) for 8 consecutive weeks during December and January and peaked at 4.8% during the week ending January 1, 2022. This peak coincided with the rise in activity of SARS-CoV-2 related to the Omicron variant. ILI activity increased again from mid-February through mid-May, but stayed below baseline. Multiple respiratory viruses were cocirculating during both periods of increasing ILI activity, and the relative contribution of influenza virus infection to ILI varied by week and location.

## Long-Term Care Facilities Surveillance

Reporting of influenza among residents of long-term care facilities^§§^ was added to the national influenza surveillance system for the 2021–22 season. The weekly percentage of facilities reporting at least one influenza-positive test result among residents ranged from 0.1% to 1.4%.

## Influenza-Associated Hospitalizations

**FIGURE 2:**
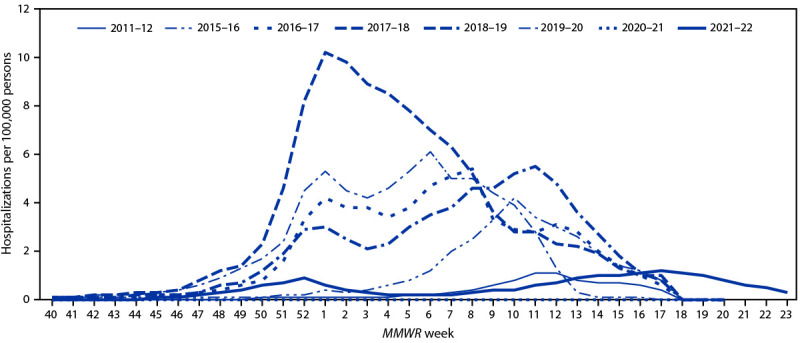
Weekly rate* of hospitalizations among patients of all ages with laboratory-confirmed influenza — United States, October–June, 2011–12 and 2015–16 to 2021–22 * Weekly rates for all seasons before the 2021–22 season reflect end-of-season rates. For the 2021–22 season, rates for recent hospital admissions are subject to reporting delays. As hospitalization data are received each week, case counts and rates are updated accordingly. Because of late season activity during the 2021–22 season, the Influenza Hospitalization Surveillance Network has extended surveillance beyond the typical end date of April 30 (*MMWR* week 17).

CDC has monitored hospitalizations associated with laboratory-confirmed influenza infections through the Influenza Hospitalization Surveillance Network (FluSurv-NET),^¶¶^ which covers approximately 9% of the U.S. population, for many years. During the 2021–22 influenza season, HHS Protect Hospitalization Surveillance,*** which consists of reports from all U.S. hospitals, was added to monitor severe illnesses requiring hospitalization in all 50 states, the District of Columbia, and U.S. territories. During October 1, 2021–June 11, 2022, a total of 5,079 laboratory-confirmed influenza-related hospitalizations were reported by FluSurv-NET sites. Activity occurred in two waves, with hospitalization rates first peaking nationally during the week ending January 1, 2022 (week 52) at 0.9 per 100,000 population and the second, slightly higher peak, during the week ending April 30, 2022 (week 17) at 1.2 per 100,000 population (Figure 2).

The overall cumulative hospitalization rate was 17.3 per 100,000 population, with the highest rate among adults aged ≥65 years (50.0), followed by children aged 0–4 years (21.9), adults aged 50–64 years (16.0), children and adolescents aged 5–17 years (9.0), and was lowest among adults aged 18–49 years (9.1). Most (96.7%) influenza-related hospitalizations were associated with influenza A viruses (98.8% of those subtyped were A[H3N2] viruses). Among patients with information about underlying conditions, 93.3% of adults and 64.8% of children and adolescents reported at least one underlying medical condition.

A total of 62,300 influenza-associated hospitalizations were reported to HHS Protect Hospitalization Surveillance during two waves of activity; the total cumulative rate was 19.0 per 100,000 population. Similar to FluSurv-NET–reported activity, nationwide, the first wave of admissions peaked during late December (week ending January 1, 2022), and the second, higher peak, occurred during mid-April (week ending April 23, 2022). Regionally, the timing of the second wave peak varied; regions 6 and 7 (Central and South Central, respectively) peaked in mid-March, regions 2, 3, and 5 (New York/New Jersey/Puerto Rico, Mid-Atlantic, and Midwest, respectively) peaked in April, regions 1, 8, 9, and 10 (New England, Mountain, West Coast, and Pacific Northwest, respectively) peaked in May, and region 4 (Southeast) peaked in early June.

## Influenza Mortality

According to the National Center for Health Statistics Mortality Surveillance System, the weekly percentage of deaths due to pneumonia, influenza, or COVID-19 remained above the epidemic threshold^†††^ during the entire 2021–22 season. Among the 387,112 deaths due to pneumonia, influenza, or COVID-19 reported during 2021–22, a total of 277,350 (71.6%) death certificates listed COVID-19 as an underlying or contributing cause of death, and 2,493 (0.6%) listed influenza, indicating that pneumonia, influenza, or COVID-19–associated mortality during 2021–22 was due primarily to COVID-19 and not influenza.

CDC monitors pediatric influenza-associated deaths through the Influenza-Associated Pediatric Mortality Surveillance System. During October 3, 2021–June 11, 2022, a total of 31 laboratory-confirmed influenza-associated pediatric deaths were reported to CDC; all were associated with an influenza A virus infection, and all of the 13 influenza A viruses with subtyping information were A(H3N2) viruses. The mean age was 6 years (range = 2 months–16 years), and 21 (67.7%) children and adolescents died after hospital admission. Among the 29 children and adolescents with a known medical history, 19 (65.5%) had at least one underlying medical condition associated with increased risk for influenza-related complications.

## Preliminary Estimates of Influenza Burden

CDC uses the cumulative rates of influenza-associated hospitalizations reported through FluSurv-NET and a mathematical model to estimate the number of persons who have symptomatic influenza illness and who had a medical visit or were hospitalized for or died from influenza (*5*). Using data available during October 1, 2021–June 11, 2022, CDC estimates that influenza virus infection resulted in 8.0–13.0 million symptomatic illnesses, 3.7–6.1 million medical visits, 82,000–170,000 hospitalizations, and 5,000–14,000 deaths in the United States.

## Discussion

Since SARS-CoV-2 emerged in the United States in early 2020, influenza activity has been lower than that seen before the pandemic. The adoption of COVID-19–related mitigation measures might have had an impact on the timing or severity of influenza activity. Compared with prepandemic influenza seasons, the 2021–22 influenza season was mild and occurred in two waves, with the second wave having a higher percentage of positive clinical laboratory test results and a higher number of hospitalizations than did the first. Influenza activity peaked later and remained at higher levels than had been reported in previous seasons in late April, May, and early June. During the 2021–22 season, peak percentage of positive influenza test results from clinical laboratories was the lowest in at least 25 years preceding the COVID-19 pandemic, and the cumulative rate of influenza-associated hospitalizations was lower than that in all but the 2011–12 season, the mildest influenza season during the 10 years before the COVID-19 pandemic. The estimate of symptomatic illnesses, medical visits, hospitalizations, and deaths caused by influenza virus infection in the United States during the 2021–22 season is also lower than estimates for any of the 10 influenza seasons preceding the pandemic. The lower level of influenza activity is not because of a decrease in testing for influenza; clinical laboratories reporting to CDC tested ≥1 million more specimens, and public health laboratories tested at least seven times as many specimens during the 2021–22 season than in any of the five seasons preceding the pandemic (2015–16 season to 2019–20 season).

The first wave of influenza activity during the 2021–22 season peaked in mid-December throughout the country, but the timing of peak activity during the second wave varied by region, ranging from mid-March to May. Notably, the second wave peaked and influenza activity remained elevated nationally later than in any previous seasonal influenza epidemic. The predominant influenza virus throughout both waves was influenza A(H3N2) virus. Most of these viruses belonged to the 3C.2a1b.2a.2 subclade and were antigenically distinct from the reference viruses representing the egg-grown and cell-grown A(H3N2) vaccine components for the 2021–22 Northern Hemisphere influenza vaccines; however, based on preliminary vaccine effectiveness estimates, persons who were vaccinated during the 2021–22 season reduced their risk for influenza illness by approximately one third.^§§§^ The recommended A(H3N2) component for the 2022–23 influenza vaccine was updated to one that belongs to the 3C.2a1b.2a.2 subclade, the subclade that predominated in the United States during the 2021–22 season. All the influenza viruses collected and tested for antiviral resistance by CDC since October 3, 2022, were susceptible to zanamivir, and the majority (>99%) were susceptible to baloxavir, oseltamivir, and peramivir.

Despite decreasing influenza activity in recent weeks, maintaining vigilance for influenza virus infections throughout the summer is important. Sporadic seasonal influenza virus infections and novel influenza A virus infections associated with exposure to swine during animal exhibitions are often reported during summer months (*6*). In addition, an ongoing outbreak of highly pathogenic avian influenza A(H5N1) virus among birds during the 2021–22 season underscores the importance that providers and persons with exposure to sick or infected birds remain attentive to any new symptoms that could be consistent with influenza virus infection (*7*). Patients with suspected novel influenza A virus should isolate at home away from household members and refrain from going to work or school until they are proven to not be infected or have recovered from their illness. Specimens from patients with suspected novel influenza A virus infection should be collected and referred to state public health departments for testing, and treatment with influenza antiviral medications should be initiated immediately. Treatment is recommended and should be initiated as soon as possible for patients with confirmed or suspected seasonal or swine influenza virus infection who have severe, complicated, or progressive illness; who require hospitalization; or who are at increased risk for influenza-associated complications (*8*). Influenza antiviral drugs are approved by the Food and Drug Administration for treatment of acute uncomplicated influenza within 2 days of illness onset and are recommended for use in the United States during the 2021–22 season. For persons aged ≥6 months, receiving a seasonal influenza vaccine each year remains the best way to protect against seasonal influenza and its potentially severe consequences.

Influenza surveillance reports for the United States are posted online weekly (https://www.cdc.gov/flu/weekly). Additional information regarding influenza viruses, surveillance, vaccines, antiviral medications, and novel influenza A infections in humans is available online (https://www.cdc.gov/flu).

**Summary**

**What is already known about this topic?**

CDC collects, compiles, and analyzes data on U.S. influenza activity and viruses.

**What is added by this report?**

The severity of the 2021–22 influenza season was low, with two waves of influenza A activity. Influenza activity continued from October 2021 through mid-June 2022, with A(H3N2) viruses predominating throughout the season. This report also describes the composition of the Northern Hemisphere 2022–23 influenza vaccine.

**What are the implications for public health practice?**

Because of the atypical timing and duration of influenza activity, providers and patients should consider influenza infection as a cause of respiratory illness. Testing for seasonal influenza and monitoring for novel viruses, especially avian A(H5N1) and swine viruses, should continue year-round. Receiving a seasonal influenza vaccine each year remains the best way to protect against seasonal influenza and its potentially severe consequences.

## Footnotes

* These authors contributed equally to this report.

† 45 C.F.R. part 46, 21 C.F.R. part 56; 42 U.S.C. Sect. 241(d); 5 U.S.C. Sect. 552a; 44 U.S.C. Sect. 3501 et seq.

§ Additional specimens initially reported as positive for a B/Yamagata lineage virus were found to be associated with recent live attenuated influenza vaccine (LAIV) receipt or upon further testing were found to be a vaccine virus. The one B/Yamagata positive specimen in this report could not be tested further by CDC to determine if it was an LAIV virus.

¶
https://www.hhs.gov/about/agencies/iea/regional-offices/index.html

** ILI is defined as fever (temperature of 100°F [37.8°C] or higher), cough, or sore throat. The case definition no longer includes “without a known cause other than influenza.”

††
https://www.cdc.gov/flu/weekly/overview.htm#anchor_1539281266932

§§
https://www.cdc.gov/flu/weekly/overview.htm#anchor_1633698456778

¶¶ FluSurv-NET conducts population-based surveillance for hospitalizations in children and adolescents aged <18 years (since the 2003–04 influenza season) and adults aged ≥18 years (since the 2005–06 influenza season) associated with laboratory-confirmed influenza. FluSurv-NET covers approximately 70 counties in the 10 Emerging Infections Program states (California, Colorado, Connecticut, Georgia, Maryland, Minnesota, New Mexico, New York, Oregon, and Tennessee) and four Influenza Hospitalization Surveillance Project states (Iowa, Michigan, Ohio, and Utah) and represents approximately 9% of the U.S. population. https://www.cdc.gov/flu/weekly/influenza-hospitalization-surveillance.htm

***
https://www.cdc.gov/flu/weekly/overview.htm#anchor_1634240269291

††† The seasonal baseline proportion of pneumonia and influenza deaths is projected using a robust regression procedure, in which a periodic regression model is applied to the observed percentage of deaths from pneumonia, influenza, or COVID-19 that were reported by the National Center for Health Statistics Mortality Surveillance System during the 5 years before the COVID-19 pandemic. The epidemic threshold is set at 1.645 SDs above the seasonal baseline.

§§§
https://www.cdc.gov/flu/spotlights/2021-2022/specific-vaccines-seniors.htm
